# Editorial: Proficiency testing in histocompatibility and immunogenetics: current status and future perspectives

**DOI:** 10.3389/fgene.2025.1619032

**Published:** 2025-06-05

**Authors:** Martin Petrek, Kelley M. K. Hitchman

**Affiliations:** ^1^ Department of Pathophysiology, Faculty of Medicine and Dentistry, Palacky University Olomouc, Olomouc, Czechia; ^2^ Departments of Immunology and Clinical & Molecular Pathology, University Hospital Olomouc, Olomouc, Czechia; ^3^ University Health, San Antonio, TX, United States; ^4^ University Health Transplant Institute, San Antonio, TX, United States; ^5^ Department of Pathology and Laboratory Medine, The University of Texas Health Science Center at San Antonio, San Antonio, TX, United States

**Keywords:** histocompatibility, quality control, HLA typing, crossmatch, chimerism, antibody identification, transplantation

Proficiency testing (PT) has been part of Histocompatibility and Immunogenetics (H&I) since the early days. Starting from spontaneous interlaboratory comparisons, it gradually moved to more elaborate quality control exercises. Immunogenetics PT finally evolved into a formalized system comprising a range of testing schemes, the rules of which have been fine-tuned and approved with the assistance of professional societies. This collection of papers presents contributions covering major areas of current H&I practice in the PT context. The authors are active immunogeneticists, mostly working as clinical laboratory scientists, but in some cases also those working in PT program development. Contributions come both from Europe and North America, and while the H&I is undoubtedly of international character, and experience and lessons learned from PT are shared, we will briefly summarise particular contributions by geographical region in the following text.

Zoet et al. describe principles of the external proficiency testing (EPT) organized by Eurotransplant; one of the oldest EPT programs (established in 1978) consisting of schemes for HLA typing including serology, for CDC crossmatching, and for HLA-specific antibody detection and identification. Voorter et al. reports on an EPT scheme run from Maastricht, the Netherlands, which is aimed namely at the high-resolution typing of HLA class I (HLA-A,-B,-C) and class II (HLA-DRB1, -DRB3,4,5, -DQA1, -DQB1, -DPA1 and -DPB1) alleles, including allelic resolution typing for HLA class I–a feature being unique. In their perspective article, d´Ath et al. highlight some of the technological and clinical milestones in HLA typing to show the history and continual evolution of EPT schemes provided by the UK NEQAS for H&I. These include not only continually evolving DNA based typing methods, but also expansion into crossover discipline application areas, as exemplified by PT on pharmacogenetic testing. The authors also emphasize the need to move an EPT service from solely covering the technical elements of the laboratory testing, to include appraisal of result interpretation and clinical advice, which is indeed the opinion shared by most stakeholders in the PT field.

While Eurotransplant, UK NEQAS and the Maastricht schemes cover multiple countries/regions, there have been national EPT schemes in Europe. Hereby, Martin et al. characterizes Spanish EPT program GECLID which, besides covering HLA typing and crossmatching, runs schemes for human platelet antigen (HPA), killer inhibitory receptor (KIR) typing, and chimerism testing. The report by Vrana et al. summarizes the 10-year experience of Czech organizers of the EPT scheme “Detection of HLA Alleles Associated with Diseases” focused on standardization, harmonization, and improvement of the overall quality of the HLA investigations for the coeliac disease diagnosis. In this regard, the relevance of population genetics for EPT is brought by Mrazek, who emphasizes the need to reflect population-based differences in disease-associated HLA alleles, distribution and linkage disequilibrium of HLA alleles in particular populations, and interpretation of the presence of less common HLA variants/haplotypes. Bogunia-Kubik et al. present a perspective on the development and organizational principles of the Polish national system of supervision and control of histocompatibility laboratories, discuss problems which may occur and suggest prospects for the future. Oguz puts proficiency testing in H&I testing again in the wider context of Quality assurance and emphasizes the importance of EPT for accreditation, e.g. by the European Federation for Immunogenetics (EFI), and reviews existing and potential EPT programs.

The opinion piece by Doxiadis and Lehmann, closing the “European” part, is a natural bridge between the contributions from the two continents as it is generally applicable. The authors (among others) endorse the digital (electronic) handling of PT data. They call for greater flexibility of PT programs in order to reflect the changing nature of the field, including s.c.“experimental” PT in cases of new methodologies They also suggest exploiting the potential of experience from PT to contribute to formulation of organisational policies, e.g. in transplant setting.

H&I EPT in North America is largely provided by the American Society of Histocompatibility & Immunogenetics (ASHI), the College of American Pathologists (CAP) and the UCLA exchange programs, with international laboratories around the globe often subscribing to these North American programs as well. These programs, and their subscribers (or participants) acknowledge that swift advances in testing, data and analytic diversity necessitate EPT diversification in the H&I space; an important consideration given that Centers for Medicare & Medicaid Services (CMS) and the Clinical Laboratory Improvement Amendments (CLIA) in the United States require high complexity laboratories to subscribe to EPT, or alternative EPT, for all methods of testing utilized.

The ASHI EPT program, as detailed by Hod-Dvorai et al., highlights EPT survey data as an essential educational tool in testing and clinical consultation, with its virtual crossmatch challenge offering global participants blinded simulation cases to form HLA-based immunologic transplant risk assessments with paired anti-HLA antibody and lymphocyte crossmatch results from ASHI EPT antibody identification and lymphocyte crossmatch surveys. The 75-year history and evolution of the CAP histocompatibility PT program is reviewed by Sullivan et al., with current and future perspectives on antibody, molecular, engraftment, parentage/relationship, disease association and drug hypersensitivity testing and analytic trajectories discussed. Zhang et al. chronicle expansion of the UCLA Cell Exchange program, founded in 1974 with a focus on international collaboration, which has transformed into a provider of HLA molecular typing, antibody, cytotoxicity, flow cytometric crossmatch and KIR gene typing challenge to over 30 countries around the globe. Authors highlight gaps in EPT provision of non-HLA antibody, eplet analytics and Swine Leukocyte Antigen (SLE) challenges. Surge in next-generation sequencing (NGS) and third-generation sequencing access and application parallel more frequent identification of novel alleles, presenting opportunities and challenges for EPT. Tran et al. highlight the frequency of NGS-based novel allele detection in British Columbia, Canada, while also providing protocols for standardization of typing confirmation, assessment of novel polymorphism impacts to HLA proteins and submission to the Immuno Polymorphism Database-Immunogenetics/HLA (IPD-IMGT/HLA). Kakodkar et al. underscore the utility of NGS sensitivity in chimerism assessment of allogenic hematopoietic stem cell transplantation (allo-HSCT) recipients and call for EPT programs to develop challenges designed to support accuracy in key cell subset chimerism testing.

All the fourteen articles collected in this unique Research Topic are a testament to EPT adaptation in the technology and data innovation wellspring of H&I, but appropriately present EPT programs with a call to action for provision of new and enhanced PT challenges. The Editors believe that the collection and/or individual reports will be useful for the truly international and global H&I community. As the field has been constantly evolving, the Immunogenetics section of the Frontiers in Genetics would be happy to continue considering contributions on the diverse topic of PT in H&I. Submissions from authors representing other/additional continents would be valuable and most welcome.

